# Reduced *Plasmodium vivax* Erythrocyte Infection in PNG Duffy-Negative Heterozygotes

**DOI:** 10.1371/journal.pone.0000336

**Published:** 2007-03-28

**Authors:** Laurin J. Kasehagen, Ivo Mueller, Benson Kiniboro, Moses J. Bockarie, John C. Reeder, James W. Kazura, Will Kastens, David T. McNamara, Charles H. King, Christopher C. Whalen, Peter A. Zimmerman

**Affiliations:** 1 Center for Global Health and Disease, Case Western Reserve University, School of Medicine, Cleveland, Ohio, United States of America; 2 Papua New Guinea Institute of Medical Research, Goroka, Papua New Guinea; 3 Department of Epidemiology and Biostatistics, Case Western Reserve University, School of Medicine, Cleveland, Ohio, United States of America; North Carolina State University, United States of America

## Abstract

**Background:**

Erythrocyte Duffy blood group negativity reaches fixation in African populations where *Plasmodium vivax* (Pv) is uncommon. While it is known that Duffy-negative individuals are highly resistant to Pv erythrocyte infection, little is known regarding Pv susceptibility among heterozygous carriers of a Duffy-negative allele (+/−). Our limited knowledge of the selective advantages or disadvantages associated with this genotype constrains our understanding of the effect that interventions against Pv may have on the health of people living in malaria-endemic regions.

**Methods and Findings:**

We conducted cross-sectional malaria prevalence surveys in Papua New Guinea (PNG), where we have previously identified a new Duffy-negative allele among individuals living in a region endemic for all four human malaria parasite species. We evaluated infection status by conventional blood smear light microscopy and semi-quantitative PCR-based strategies. Analysis of a longitudinal cohort constructed from our surveys showed that Duffy heterozygous (+/−) individuals were protected from Pv erythrocyte infection compared to those homozygous for wild-type alleles (+/+) (log-rank tests: LM, *p* = 0.049; PCR, *p* = 0.065). Evaluation of Pv parasitemia, determined by semi-quantitative PCR-based methods, was significantly lower in Duffy +/− vs. +/+ individuals (Mann-Whitney U: *p* = 0.023). Overall, we observed no association between susceptibility to *P. falciparum* erythrocyte infection and Duffy genotype.

**Conclusions:**

Our findings provide the first evidence that Duffy-negative heterozygosity reduces erythrocyte susceptibility to Pv infection. As this reduction was not associated with greater susceptibility to Pf malaria, our *in vivo* observations provide evidence that Pv-targeted control measures can be developed safely.

## Introduction

In PNG, malaria is caused by *P. vivax* (Pv), *P. falciparum* (Pf), *P. malariae* (Pm) and *P. ovale* (Po), and endemic in regions below 1600 meters elevation [1]. Individual infections with multiple *Plasmodium* species (mixed infections) are common, and there is growing interest in the potential that cross-species regulatory mechanisms control parasitemia and malarial disease [2]–[5].

Ministry of Health statistics show that malaria represents the most significant public health burden in PNG [6]. Moreover the overlapping occurrence of erythrocyte polymorphisms in malarious regions of PNG [7]–[10] suggests that malaria exerts significant selective pressure on the human population as proposed for other malaria-endemic regions of the world [11]. In large part it is presumed that selective pressure by malaria is associated with Pf because high rates of severe falciparum malaria mortality are observed in Africa. Although the selective burden attributable to Pv has been more difficult to substantiate, recent studies have shown evidence for severe vivax malaria [12], [13]. A convergence of human genetics and *Plasmodium* species interactions observed previously in Vanuatu led Williams *et al*. [14] to the controversial hypothesis that α^+^-thalassemia predisposes young children to Pv infection and that early exposure to this more “benign” species reduced the risk of severe falciparum malaria. Under this hypothesis host genetic adaptations that reduce the risk of Pv would not be favored to arise in regions that were co-endemic for Pf.

It was surprising then that our earlier studies in PNG discovered the same single nucleotide polymorphism (SNP) in the Duffy gene promoter observed on the African Duffy negative FY*B^null^ (B−) allele on the FY*A allele [15] in a Melanesian population experiencing year-round hyperendemic exposure to both Pv and Pf [16] (Duffy nomenclature summarized in Methods). In our original study, we identified 23 heterozygous carriers of this FY*A^null^ allele (A−) and no homozygous negative (A−/A−) individuals [15]. Duffy antigen expression on erythrocytes from A+/A− individuals was reduced by 50% compared to levels expressed on erythrocytes from A+/A+ individuals [15]. In *in vitro* assays, erythrocytes from A+/A− donors (compared to A+/A+ donors) formed significantly fewer rosettes when mixed with COS-7 cell transfectants expressing the Pv Duffy binding protein [17]. These preliminary findings suggested that reduced expression of the Duffy blood group antigen on red blood cells would decrease susceptibility to Pv blood-stage infection. Here, through multiple epidemiologic surveys evaluating malaria infection status at different time-points, we present evidence that the Duffy A+/A− genotype is associated with reduced susceptibility to Pv blood-stage infection *in vivo* while not affecting the risk of Pf infections.

## Methods

### Study design and human subjects

We conducted two sets of serial cross-sectional surveys to estimate malaria prevalence in the Wosera, East Sepik Province, Papua New Guinea. The first set of seven prevalence surveys was conducted monthly from July 1998 to January 1999 in six villages in the Wosera (cumulative population 1845), where the Papua New Guinea Institute of Medical Research (PNGIMR) has performed numerous malaria epidemiologic surveys [16]. Participation was voluntary and village residents were free to choose when or if they participated in a survey. During the survey period, 1,689 (91.5%) residents from the six villages participated in at least one blood draw; 6,752 blood samples were collected. From these surveys, we constructed a longitudinal cohort of 467 children (2531 observations) who were under 15 years of age and who had attended four or more of the monthly malaria prevalence surveys. To control for the potential confounding effects of age, sex, and village of residence, we matched 11 children (58 observations) who were A+/A− with 33 children (167 observations) who were A+/A+ in a ratio of 1∶3 on these characteristics.

A second set of four malaria prevalence surveys was conducted at approximately 6-month intervals from August 2001 to June 2003 in 29 villages of the Wosera [18]. At the completion of these serial cross-sectional surveys, 8,793 (approximately 67.6%) residents participated in at least one blood draw; 16,209 blood samples were collected [18]. In each set of cross-sectional surveys, we compared susceptibility to Pv and other *Plasmodium* species blood-stage infections to Duffy genotype.

Ethical approval for these studies and procedures for oral/written informed consent were obtained from the Medical Research and Advisory Council of Papua New Guinea, the Institutional Review Board for Human Investigation of Case Western Reserve University and University Hospitals of Cleveland, OH, and the National Institute of Allergy and Infectious Diseases, International Clinical Studies Review Committee (ICSRC).

### Sample collection and DNA extraction

Samples were collected in K^+^-EDTA Vacutainer™ tubes, and stored at −20°C until DNA extraction could be performed. DNA was extracted from whole blood (200 µL) using QIAamp 96 spin blood kits (QIAGEN, Valencia, CA).

### Malaria Diagnosis

Thick/thin smears were prepared by standard procedures, stained with 4% Giemsa and examined by PNGIMR-trained microscopists under oil-immersion (100×). Parasite species and species-specific densities were identified and recorded while counting the number of microscope fields containing 200 leukocytes as described previously [16]. Conformational diagnosis of all four human *Plasmodium* parasite species was performed by a post-PCR ligase detection reaction/fluroescent microsphere assay (LDR-FMA) [19], [18], [20]. Finally, we performed real-time quantitative PCR (RTQ-PCR) analysis of Pv blood-stage infection by methods described previously [21].

### Duffy (*FY*) nomenclature

A single nucleotide polymorphism (SNP) in the Duffy gene (*FY*) corrupts the GATA-1 transcription factor binding site regulating erythroid Duffy antigen expression specifically (−33 T→C [22]; numbering based on Iwamoto et al., 1996 [23]). A second SNP responsible for a glycine (G) to aspartic acid (D) substitution [24] at amino acid 42 underlies Fy^a^ and Fy^b^ antigenic variation. Combinations of these two SNP lead to FY*A (A+), FY*A^null^ (A−), FY*B (B+) and FY*B^null^ (B−) alleles [25].

### PCR-based Genotyping

A 329 bp fragment inclusive of the GATA-1 SNP in the *FY* gene was PCR amplified as previously described [15] using an upstream primer FY277(+) 5′-CAGGAAGACCCAAGGCCAG-3′, and downstream primer FY605(−) 5′-CCATGGCACCGTTTGGTTCAGG-3′; two-step thermocycling conditions 30 seconds at 94°C and 45 seconds at 68°C were repeated for 40 cycles. Restriction fragment length polymorphism (RFLP) was used to distinguish between the 77 bp *FY+* (−33T) and 65 bp *FY−* (−33C) allelic fragments using the restriction endonuclease *Sty*I following recommended protocol (New England BioLabs, Beverly, MA). Restriction fragments were separated by electrophoresis on 4% 1xTBE agarose gels (5∶1 GTG∶NuSieve∶LE, FMC BioProducts, Rockland, ME), stained with a 1∶10,000 dilution of SYBR® Gold (Molecular Probes, Eugene, OR), and visualized on a Storm® 860 Scanner (Molecular Dynamics, Sunnyvale, CA). Genotyping assays for the *FYA* vs *FYB* SNP [15], the 27 bp deletion in erythrocyte membrane protein band 3 (*SLC4A1Δ27*) [9], the glycophorin C exon 3 deletion (*GYPCΔex3*) [9] underlying Gerbich negativity, and the α-globin deletions [26] associated with *α−*thalassemia were performed using methods previously described.

### Statistical Analysis

The primary aim of the analysis was to compare the susceptibility to Pv blood-stage infection according to Duffy genotype. Further analyses compared susceptibility to all other *Plasmodium* species by Duffy genotype. To address this aim, we compared the incidence rates of blood-stage infection (estimated as infections per 100 person-months of observation) among children who were A+/A− with children who were A+/A+ using the rate ratio and 95% confidence intervals [27]. We estimated the proportion who escaped blood-stage infection during the observation period using Kaplan-Meier methods and compared curves using the log-rank test. To assess the potential effect of correlation between household members, we calculated the intra-class correlation coefficient [28] for household infection and found that it was ≤0.10, indicating household interdependence did not greatly affect study statistics. Finally, we evaluated Pv parasite density and mean fluorescent signal intensity (a measure that correlates with the intensity of Pv infection) over time in an analysis of repeated measures using generalized estimating equations [29]. To adjust for potential confounding effects, we included age, sex, village, and genotype information as time-independent variables and Pf infection status and season as time-dependent variables. Final models were selected using fit as a criterion in a series of iterations [29]–[31] in which the correlation matrix and mean distribution were systematically varied. In the latter surveys, differences in mean Pv and Pf fluorescent signal intensity between A+/A− and A+/A+ children were also compared using Mann-Whitney U tests.

We performed all statistical analyses using SAS® software (SAS Institute, version 9.1, Cary, NC).

## Results and Discussion

From surveys conducted since July 1998, our primary objective was to investigate the relationship between the Duffy A− allele and susceptibility to Pv blood-stage infection. For these studies we performed genotyping on DNA samples from 9,092 of the 13,000 Wosera residents (69.9%) to characterize the distribution and frequency of the A− allele and investigate whether this new allele alters susceptibility to Pv and thereby confers some fitness advantage. We identified 223 Duffy A+/A− individuals and no A−/A− (Duffy-negative) individuals (Duffy A− allele frequency = 0.012). The distribution of A+/A− individuals was not random within the Wosera villages and the proportion of A− carriers within the villages ranged from 0 to 13.0%. Within the community, overall infection prevalence by the four human *Plasmodium* species parasites in recent surveys was similar to that reported previously [32], and was observed to vary depending on diagnostic methodology sensitivity (blood smear light microscopy [LM], LDR-FMA): Pv–10.4%, 27.1%; Pf–22.3%, 32.9%; Pm–4.2%, 12.4%; Po–0.2%, 5.5% [18]. Mixed *Plasmodium* species infections were observed in 2.4% (LM) or 16.8% (LDR-FMA) of individuals [18].

Our study setting in PNG is therefore distinct from African populations. In comparison to vivax-hypoendemic Africa where Duffy negative (B−/B−) homozygosity predominates and confers complete resistance to Pv erythrocyte infection, reduced Duffy erythrocyte antigen expression in PNG heterozygotes (A+/A−) may significantly reduce natural *in vivo* susceptibility to blood-stage Pv infection in vivax-hyperendemic PNG. To test this hypothesis, we analyzed data from two different epidemiologic studies in the Wosera.

**Figure 1:**
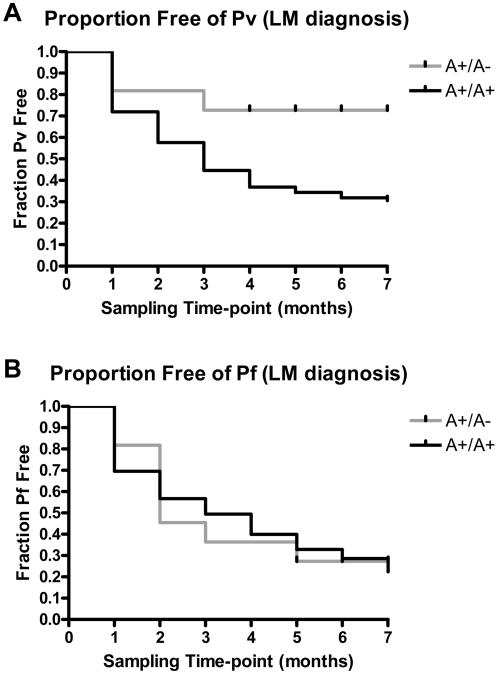
Susceptibility to blood-stage infection during monthly prevalence surveys (1998–99); unmatched longitudinal analyses. Diagnosis of Pv and Pf erythrocyte infection was performed by light microscopy (LM) [16]. Light gray line represents A+/A− (n = 11) and dark gray line represents A+/A+ (n = 453). A, Cumulative Pv erythrocyte infection in Duffy A+/A− vs A+/A+ individuals in unmatched analyses (*p* = 0.026; log-rank test). B, Cumulative Pf erythrocyte infection in Duffy A+/A− vs A+/A+ individuals in unmatched analyses (*p* = 0.875; log-rank test).

First, in an unmatched longitudinal cohort analysis (July 1998 to January 1999), we compared the rate of Pv blood-stage infection in 11 A+/A− (age range 2 to 13; mean age 8) and 456 A+/A+ (age range 1 to 14; mean age 7) children under 15 years of age who attended at least four of the monthly malaria prevalence surveys. Overall we found that the rate of Pv infection determined by conventional LM analysis was 52.0% lower in A+/A− compared with A+/A+ children (rate ratio = 0.48, 95% confidence interval (CI): 0.24, 0.96; 12.1 vs. 25.2 infections per 100 person months of observation). When we estimated the proportion of children who escaped Pv blood-stage infection during the survey period, we found that a greater proportion of A+/A− heterozygotes escaped Pv infection (72.7%) during the 7-month survey compared with A+/A+ homozygotes (30.6%) (log-rank test, *p* = 0.026; hazard ratio 0.336, 95% CI: 0.198, 0.901; Figure 1A). Using the same cohort of children, we also estimated the proportion of children who escaped Pf blood-stage infection during the survey period and found no differences between A+/A− heterozygotes (27.3%) and A+/A+ homozygotes (22.5%) (log-rank test, *p* = 0.875; hazard ratio 1.051, 95% CI: 0.471, 2.420; Figure 1B). In the longitudinal analysis adjusting for Pf co-infection and correlations over time, we found that children with the A+/A− genotype had at least a 2-fold lower mean Pv parasite density based on LM than children with the A+/A+ genotype, but this difference did not reach statistical significance (*p* = 0.16).

**Figure 2:**
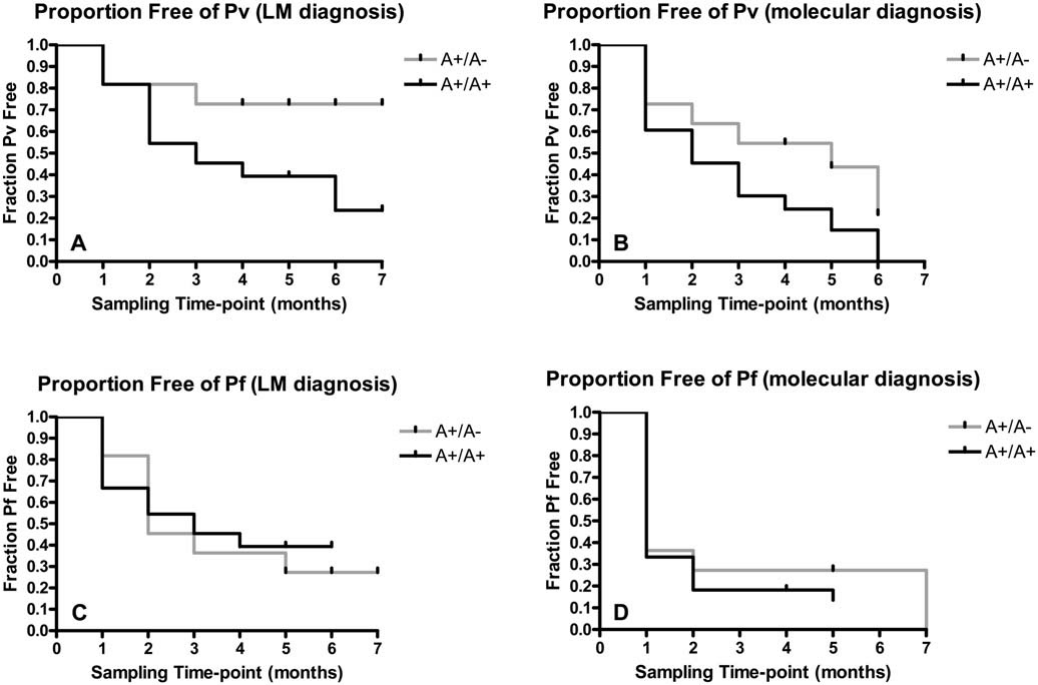
Susceptibility to blood-stage infection during monthly prevalence surveys (1998–99); matched longitudinal analyses. Evaluation of cumulative Pv and Pf erythrocyte infection was performed among Duffy A+/A− vs A+/A+ individuals matched by age, sex, and village of residence. A, Diagnosis of Pv erythrocyte infection was performed by light microscopy (LM) (*p* = 0.049; log-rank test) [16]. B, Diagnosis of Pv erythrocyte infection was performed by LDR-FMA (*p* = 0.065; log-rank test) [20]. C, Diagnosis of Pf erythrocyte infection was performed by LM (*p* = 0.675; log-rank test). D, Diagnosis of Pf erythrocyte infection was performed by LDR-FMA (*p* = 0.453; log-rank test). In A through D, light gray lines represent A+/A− (n = 11) and dark gray lines represent A+/A+ (n = 33).

To adjust for age, sex, and village of residence, we matched 11 A+/A− with 33 A+/A+ children under 15 years of age (age range 2 to 13.2; mean age 8.3) who attended at least four of the 1998–1999 cross-sectional malaria prevalence surveys. In a longitudinal analysis, we observed that A+/A− compared to A+/A+ was associated with a risk reduction of 46.0% (rate ratio = 0.54, 95% CI: 0.25, 1.13; 12.1 vs. 22.6 infections per 100 person months of observation. The proportion of A+/A− children who escaped Pv blood-stage infection (72.7%) was greater than the proportion of A+/A+ children (23.6%) (log-rank test, *p* = 0.049, hazard ratio 0.349, 95% CI: 0.146, 0.995; Figure 2A). Given the widely recognized challenges encountered in the reproducibility of malaria LM diagnosis [5], we evaluated each child's malaria status at each time point by PCR [20]. By the more sensitive test we again observed that A+/A− heterozygotes escaped Pv erythrocyte infection more successfully (21.8%) than the A+/A+ homozygotes (0%) over the 7-month survey (log-rank test, *p* = 0.065, hazard ratio 0.531, 95% CI: 0.188, 1.051; Figure 2B). We also estimated the proportion of children who escaped Pf blood-stage infection during the monthly prevalence survey period using data from both LM and PCR-based diagnostic techniques, we found no differences between A+/A− heterozygotes (27.3% LM; 0% PCR) and A+/A+ homozygotes (39.4% LM; 13.6% PCR) (LM: log-rank test, *p* = 0.675; hazard ratio 1.167, 95% CI: 0.468, 3.225; Figure 2C) (PCR: log-rank test, *p* = 0.453; hazard ratio 0.836, 95% CI: 0.203, 2.035; Figure 2D).

**Figure 3:**
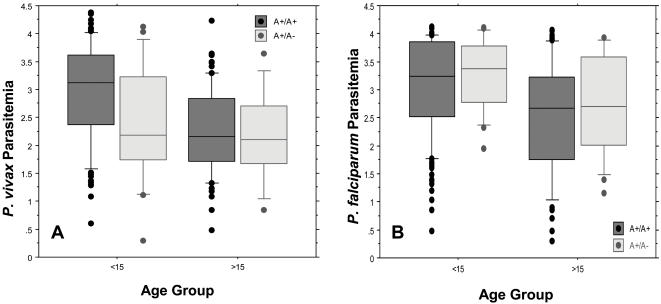
Semi-quantitative LDR-FMA Diagnosis of Pv and Pf infections split by Duffy Genotype and Age Group. A, Comparison of Pv fluorescent signal intensity (log_10_-transformed) of individuals who had a Pv-positive blood sample split by Duffy genotype (matched on age, sex, village of residence, and survey period). For children less than 15 years of age, A+/A+ (n = 105) mean fluorescent signal intensity = 2.96, A+/A− (n = 18) mean fluorescent signal intensity = 2.37 (Mann-Whitney *P* = 0.023). For individuals over 15 years, A+/A+ (n = 66) mean fluorescent signal intensity = 2.22, A+/A− (n = 15) mean fluorescent signal intensity = 2.16 (Mann-Whitney *P* = 0.86). B, Comparison of Pf fluorescent signal intensity (log_10_-transformed) of individuals who had a Pf-positive blood sample split by Duffy genotype (matched on age, sex, village of residence, and survey period). For children less than 15 years of age, A+/A+ (n = 146) mean fluorescent signal intensity = 3.08, A+/A− (n = 17) mean fluorescent signal intensity = 3.22 (Mann-Whitney *P* = 0.742). For individuals over 15 years, A+/A+ (n = 89) mean fluorescent signal intensity = 2.51, A+/A− (n = 22) mean fluorescent signal intensity = 2.69 (Mann-Whitney *P* = 0.529).

Recognizing that our observations in the initial set of surveys were based on a limited number of heterozygous children, we compared the intensity of Pv infections by age (Figure 3) in a subset of 467 children from the serial cross-sectional surveys of 29 Wosera villages (August 2001 to June 2003). Here, among children under 15 years of age, we observed that the mean Pv fluorescent signal intensity (corresponds with parasitemia [20]) was significantly lower among A+/A− individuals (µ = 2.37, log_10_ transformed) compared to A+/A+ individuals (µ = 2.96) (Mann-Whitney U*: p* = 0.023) (Figure 3A) This difference in the mean Pv fluorescent signal intensity represents a 4-fold lower signal intensity among heterozygous children. This reduction in parasitemia was similar to that observed between younger (µ = 2.81) compared to older A+/A+ individuals (µ = 2.25) (Mann-Whitney U: *p*<0.0001). These results suggest that the A+/A− genotype confers a level of protection against Pv blood stage infection that would otherwise be attributable to acquired Pv-specific immunity. Interestingly, this difference in mean Pv parasitemia conferred by A+/A− was no longer observed in individuals over 15 years of age (µ = 2.22 in A+/A+ vs. µ = 2.16 in A+/A−; Mann-Whitney U: *p*<0.86). Finally, when these studies were performed following a real-time quantitative PCR strategy to estimate Pv parasitemia, results between molecular diagnostic strategies (LDR-FMA to RTQ-PCR) were highly concordant (R^2^ = 0.620, *p*<0.0001), and comparisons between Pv susceptibility, Duffy genotype and age were identical to those reported above. In an analysis of repeated measures using generalized estimating equations to estimate the impact of the Duffy genotype on the mean Pv fluorescent signal intensity and adjust for Pf co-infection and correlations over time, we found that children with the A+/A− genotype had a 1.25-fold lower mean Pv parasite density based on LDR-FMA than children with the A+/A+ genotype (*p* = 0.017). These findings are consistent with our evaluations on infection prevalence and infection intensity showing that a reduced risk of Pv blood-stage infection was associated with the A+/A− genotype.

Because some studies have suggested that Pv infection reduces the severity of Pf infection, we were interested in determining whether Duffy genotype influenced susceptibility to Pf blood stage infection. In additional comparisons between children under and 15 years and older individuals (Figure 3B), we observed no difference in the mean Pf fluorescent signal intensity between A+/A+ and A+/A− individuals (under 15 years–3.08 vs. 3.22, Mann-Whitney-U: *p* = 0.742; 15 years and older–2.51 vs. 2.69, Mann-Whitney-U: *p* = 0.648). Similar to results for Pv parasitemia, we did observe that older individuals had lower Pf parasitemia than younger individuals. Our results suggest, therefore, that while we observed a reduction in Pv parasitemia in Duffy A+/A− compared to A+/A+ individuals, we did not observe that a reciprocal increase in Pf parasitemia was associated with Duffy A+/A−.

Whether these results offer insight into events of human evolution responsible for fixation of the Duffy B− allele in African populations would be difficult to substantiate. What is intriguing about our observations in a region of PNG endemic for both Pv and Pf is the juxtaposition between a significant reduction in Pv risk in A+/A− children and proposals that Pv infection reduces severity of falciparum malaria [14], [33]–[35], [3]. If these proposals were correct, we might expect that A+/A− individuals would experience increased morbidity and mortality resulting from *P. falciparum* infection and therefore reduced fitness. In contrast to this expectation the Duffy A− allele has been consistently inherited over at least four generations within the Wosera community, and the frequency of this new PNG allele has risen above 6.5% within some villages. These observations imply that Duffy A− confers some selective advantage within the population. As it is clear that vivax malaria can cause significant morbidity [12], [13], our observations in the Wosera would suggest that A+/A− heterozygosity may in some way be protective against vivax-related mortality. While our data do not rule out an increased risk of Pf illness in A+/A− individuals, it is reassuring that we did not detect any effect on risk of Pf infections and density of Pf infections, given that risk and degree of malaria illness is related to levels of parasitemia [36].

It is important to acknowledge that we have yet to identify a homozygous Duffy negative (A−/A−) individual in PNG and, based on a review of the Wosera Demographic Surveillance database, we have not yet identified parental partners who are both A+/A− heterozygous. Thus, it is difficult to predict, based on allele frequency, when we should expect to identify Duffy negative individuals in the Wosera. This data alone, however, cannot be used to rule out the possibility that the A−/A− homozygous genotype might be associated with significant negative fitness and constrain the Duffy A− allele to the role of a balanced polymorphism [37] in PNG.

When assessing the potential of Pv as a cause of human genetic adaptation, it is important to remember that previous characterization of Pv as a “benign” human malaria parasite are based upon data collected from adults receiving malaria therapy [38], and may have little relevance to vivax malarial disease experienced in children. Therefore, it will be important for future studies to compare susceptibility to clinical malaria between Duffy A+/A− vs. A+/A individuals in relation to Pv and Pf, in single and mixed species infections. Our results also provide new evidence that limiting access of the Pv merozoite to the Duffy antigen will reduce susceptibility to vivax blood-stage infection. Overall, as we do not observe a significant increase in Pf parasitemia in the Duffy A+/A− individuals tested, our observations suggest that efforts to develop Pv-specific vaccines should be pursued, and emphasize that strategies to target the Pv Duffy binding protein should be strongly considered.
